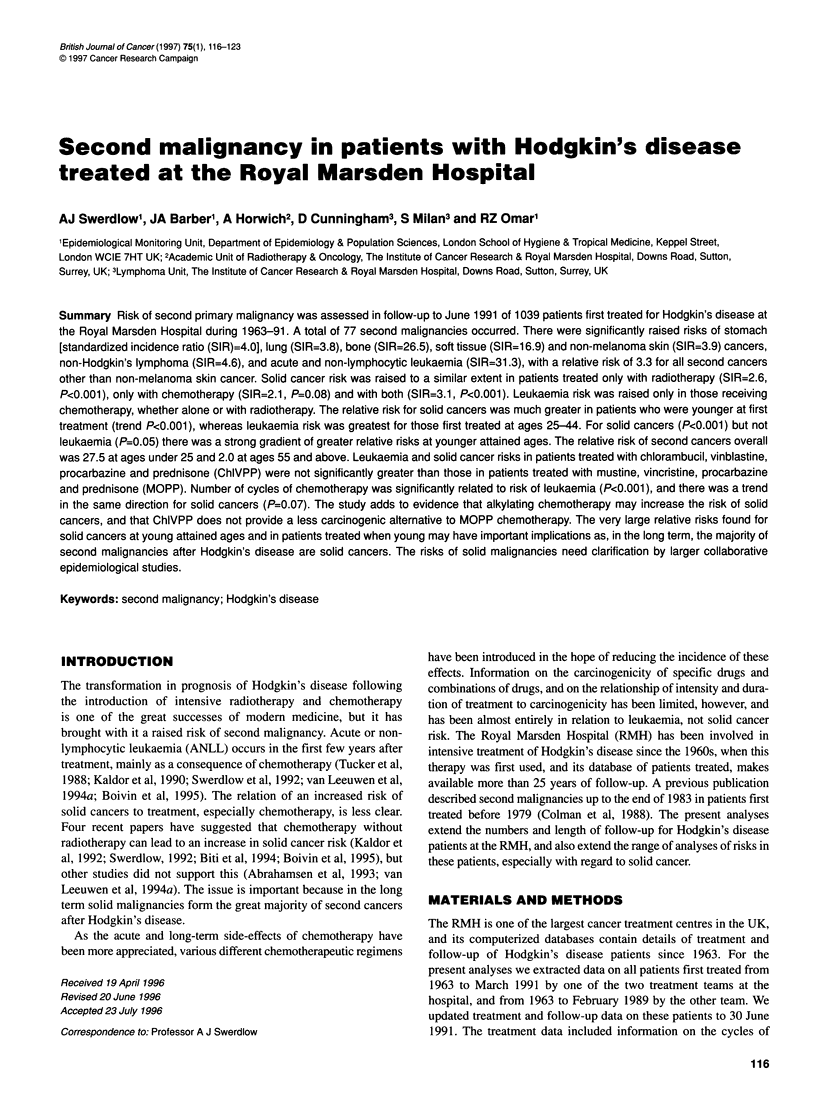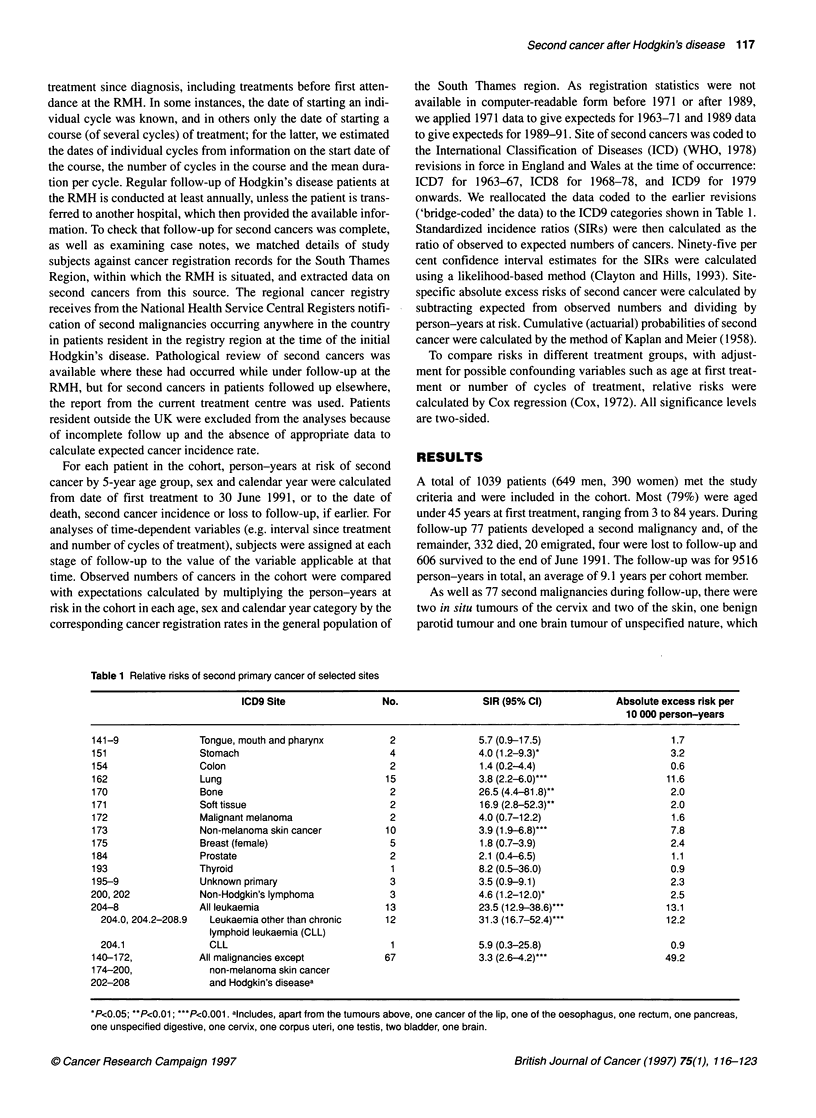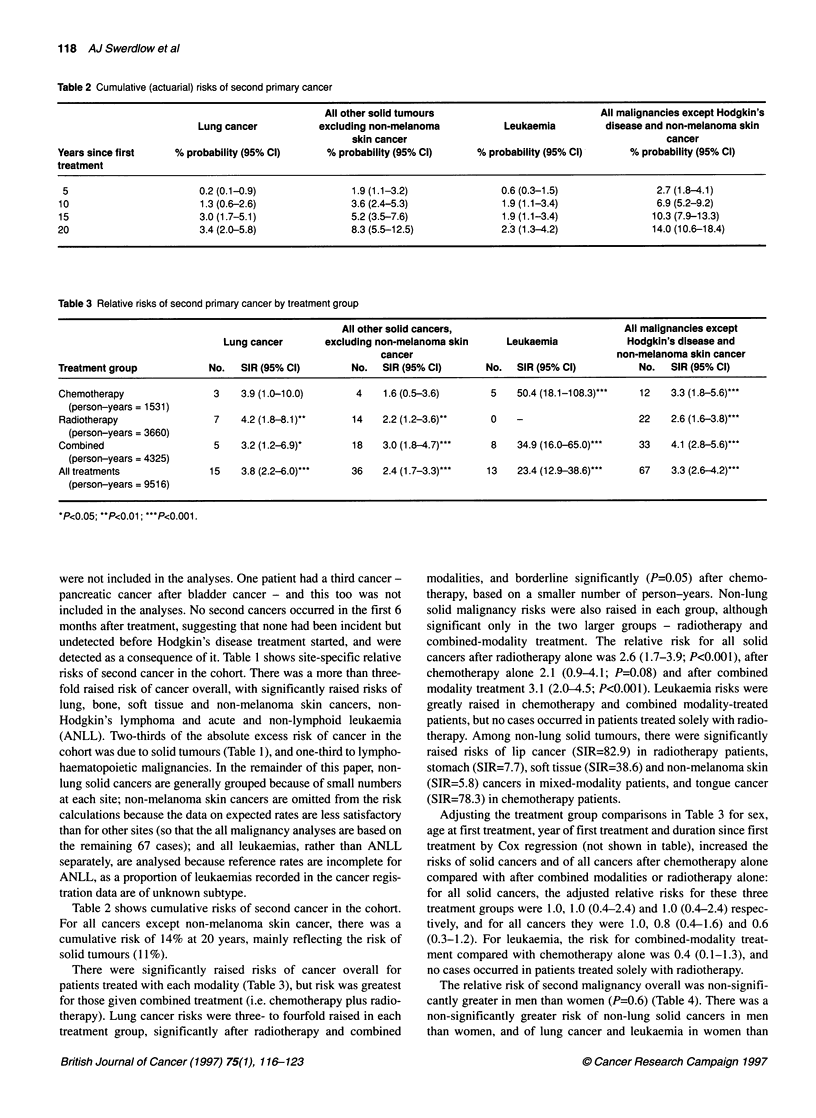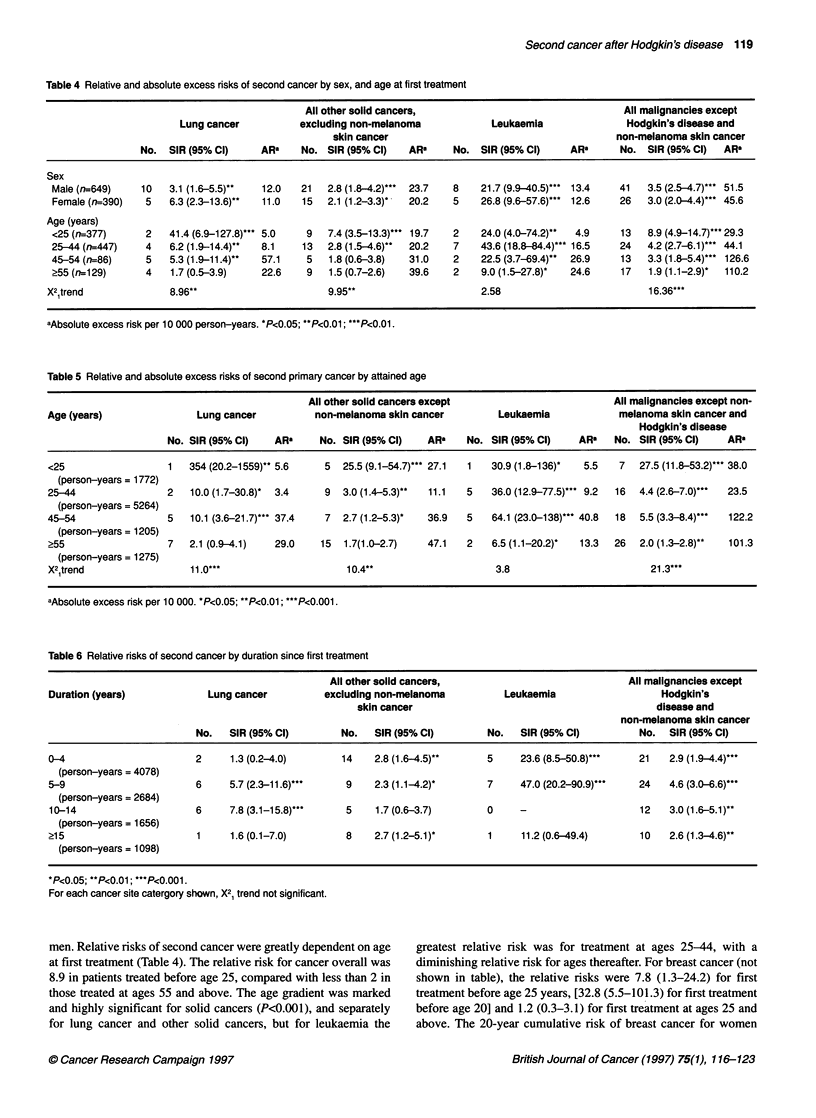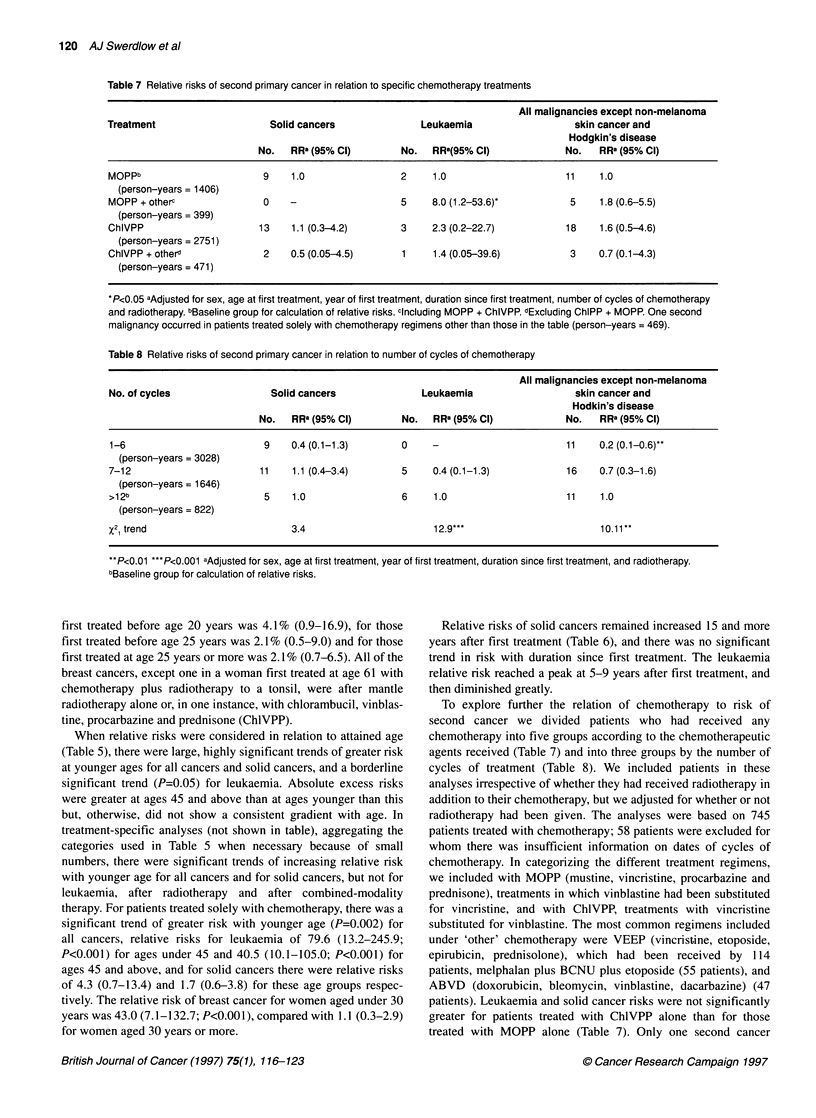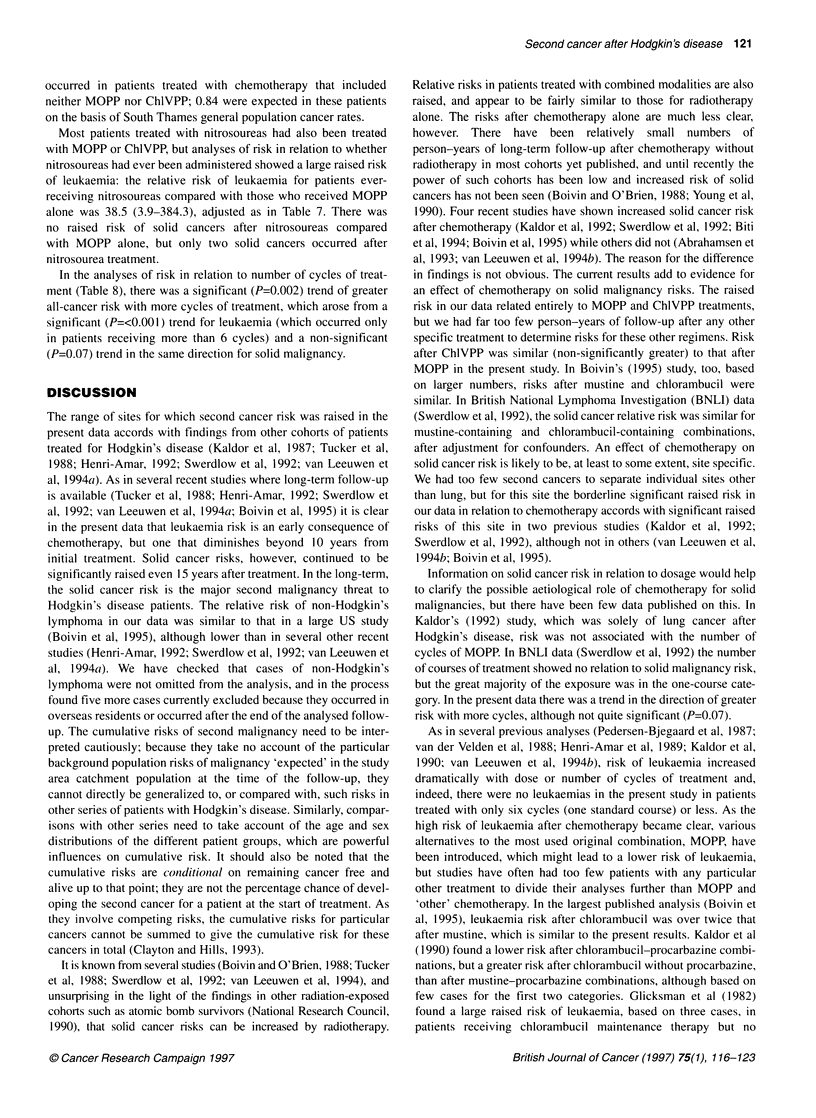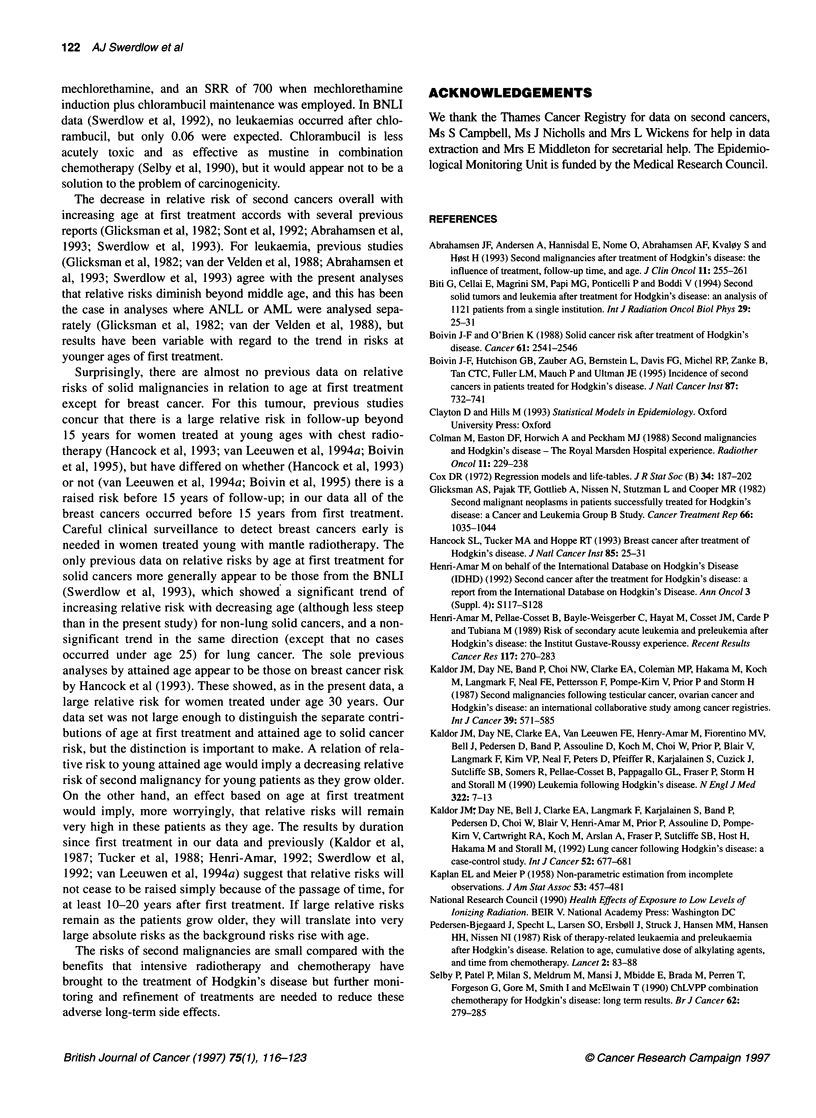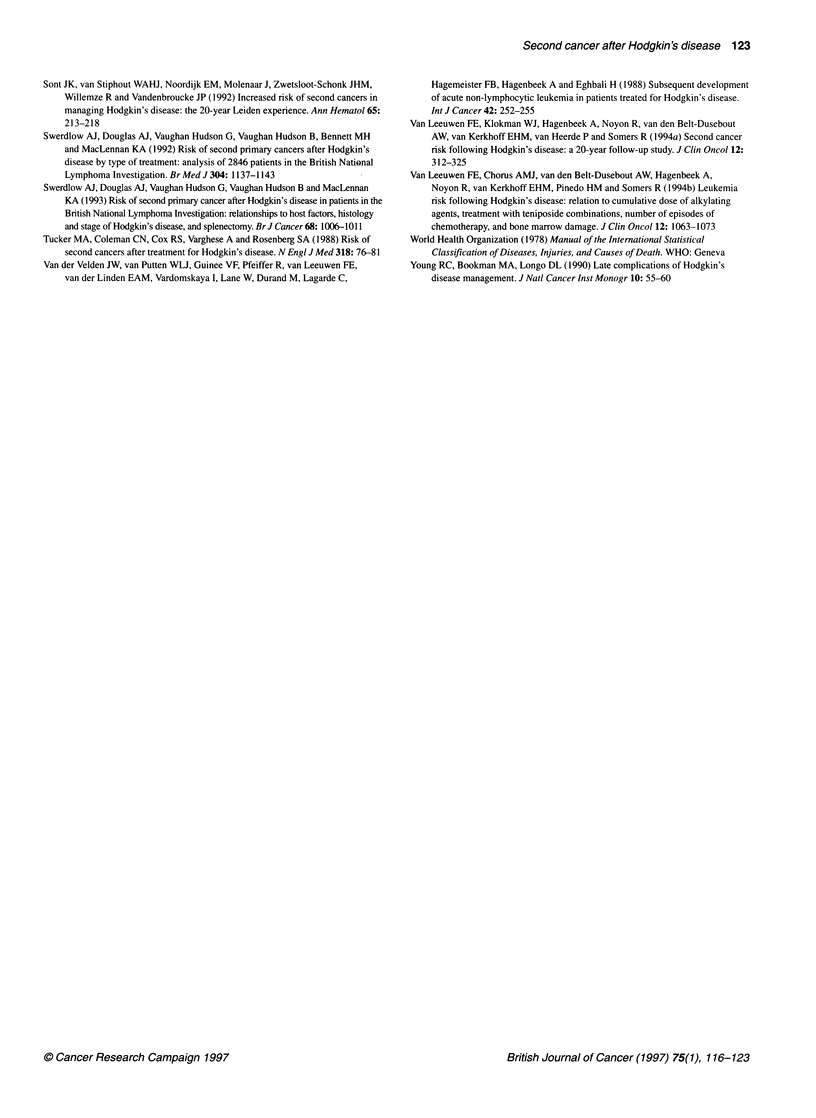# Second malignancy in patients with Hodgkin's disease treated at the Royal Marsden Hospital.

**DOI:** 10.1038/bjc.1997.19

**Published:** 1997

**Authors:** A. J. Swerdlow, J. A. Barber, A. Horwich, D. Cunningham, S. Milan, R. Z. Omar

**Affiliations:** Epidemiological Monitoring Unit, Department of Epidemiology & Population Sciences, London School of Hygiene & Tropical Medicine, UK.

## Abstract

Risk of second primary malignancy was assessed in follow-up to June 1991 of 1039 patients first treated for Hodgkin's disease at the Royal Marsden Hospital during 1963-91. A total of 77 second malignancies occurred. There were significantly raised risks of stomach [standardized incidence ratio (SIR)=4.0], lung (SIR=3.8), bone (SIR=26.5), soft tissue (SIR=16.9) and non-melanoma skin (SIR=3.9) cancers, non-Hodgkin's lymphoma (SIR=4.6), and acute and non-lymphocytic leukaemia (SIR=31.3), with a relative risk of 3.3 for all second cancers other than non-melanoma skin cancer. Solid cancer risk was raised to a similar extent in patients treated only with radiotherapy (SIR=2.6, P<0.001), only with chemotherapy (SIR=2.1, P=0.08) and with both (SIR=3.1, P<0.001). Leukaemia risk was raised only in those receiving chemotherapy, whether alone or with radiotherapy. The relative risk for solid cancers was much greater in patients who were younger at first treatment (trend P<0.001), whereas leukaemia risk was greatest for those first treated at ages 25-44. For solid cancers (P<0.001) but not leukaemia (P=0.05) there was a strong gradient of greater relative risks at younger attained ages. The relative risk of second cancers overall was 27.5 at ages under 25 and 2.0 at ages 55 and above. Leukaemia and solid cancer risks in patients treated with chlorambucil, vinblastine, procarbazine and prednisone (ChlVPP) were not significantly greater than those in patients treated with mustine, vincristine, procarbazine and prednisone (MOPP). Number of cycles of chemotherapy was significantly related to risk of leukaemia (P<0.001), and there was a trend in the same direction for solid cancers (P=0.07). The study adds to evidence that alkylating chemotherapy may increase the risk of solid cancers, and that ChlVPP does not provide a less carcinogenic alternative to MOPP chemotherapy. The very large relative risks found for solid cancers at young attained ages and in patients treated when young may have important implications as, in the long term, the majority of second malignancies after Hodgkin's disease are solid cancers. The risks of solid malignancies need clarification by larger collaborative epidemiological studies.


British Journal of Cancer (1997) 75(1), 116–123
© 1997 Cancer Research Campaign

### Second malignancy in patients with Hodgkin's disease treated at the Royal Marsden Hospital

**AJ Swerdlow[1], JA Barber[1], A Horwich[2], D Cunningham[3], S Milan[3] and RZ Omar[1]**

[1]Epidemiological Monitoring Unit, Department of Epidemiology & Population Sciences, London School of Hygiene & Tropical Medicine, Keppel Street, London WCIE 7HT UK; [2]Academic Unit of Radiotherapy & Oncology, The Institute of Cancer Research & Royal Marsden Hospital, Downs Road, Sutton, Surrey, UK; [3]Lymphoma Unit, The Institute of Cancer Research & Royal Marsden Hospital, Downs Road, Sutton, Surrey, UK

**Summary** Risk of second primary malignancy was assessed in follow-up to June 1991 of 1039 patients first treated for Hodgkin's disease at the Royal Marsden Hospital during 1963–91. A total of 77 second malignancies occurred. There were significantly raised risks of stomach [standardized incidence ratio (SIR)=4.0], lung (SIR=3.8), bone (SIR=26.5), soft tissue (SIR=16.9) and non-melanoma skin (SIR=3.9) cancers, non-Hodgkin's lymphoma (SIR=4.6), and acute and non-lymphocytic leukaemia (SIR=31.3), with a relative risk of 3.3 for all second cancers other than non-melanoma skin cancer. Solid cancer risk was raised to a similar extent in patients treated only with radiotherapy (SIR=2.6, $P<0.001$), only with chemotherapy (SIR=2.1, $P=0.08$) and with both (SIR=3.1, $P<0.001$). Leukaemia risk was raised only in those receiving chemotherapy, whether alone or with radiotherapy. The relative risk for solid cancers was much greater in patients who were younger at first treatment (trend $P<0.001$), whereas leukaemia risk was greatest for those first treated at ages 25–44. For solid cancers ($P<0.001$) but not leukaemia ($P=0.05$) there was a strong gradient of greater relative risks at younger attained ages. The relative risk of second cancers overall was 27.5 at ages under 25 and 2.0 at ages 55 and above. Leukaemia and solid cancer risks in patients treated with chlorambucil, vinblastine, procarbazine and prednisone (ChlVPP) were not significantly greater than those in patients treated with mustine, vincristine, procarbazine and prednisone (MOPP). Number of cycles of chemotherapy was significantly related to risk of leukaemia ($P<0.001$), and there was a trend in the same direction for solid cancers ($P=0.07$). The study adds to evidence that alkylating chemotherapy may increase the risk of solid cancers, and that ChlVPP does not provide a less carcinogenic alternative to MOPP chemotherapy. The very large relative risks found for solid cancers at young attained ages and in patients treated when young may have important implications as, in the long term, the majority of second malignancies after Hodgkin's disease are solid cancers. The risks of solid malignancies need clarification by larger collaborative epidemiological studies.

**Keywords:** second malignancy; Hodgkin's disease

### INTRODUCTION

The transformation in prognosis of Hodgkin's disease following the introduction of intensive radiotherapy and chemotherapy is one of the great successes of modern medicine, but it has brought with it a raised risk of second malignancy. Acute or non-lymphocytic leukaemia (ANLL) occurs in the first few years after treatment, mainly as a consequence of chemotherapy (Tucker et al, 1988; Kaldor et al, 1990; Swerdlow et al, 1992; van Leeuwen et al, 1994*a*; Boivin et al, 1995). The relation of an increased risk of solid cancers to treatment, especially chemotherapy, is less clear. Four recent papers have suggested that chemotherapy without radiotherapy can lead to an increase in solid cancer risk (Kaldor et al, 1992; Swerdlow, 1992; Biti et al, 1994; Boivin et al, 1995), but other studies did not support this (Abrahamsen et al, 1993; van Leeuwen et al, 1994*a*). The issue is important because in the long term solid malignancies form the great majority of second cancers after Hodgkin's disease.

As the acute and long-term side-effects of chemotherapy have been more appreciated, various different chemotherapeutic regimens have been introduced in the hope of reducing the incidence of these effects. Information on the carcinogenicity of specific drugs and combinations of drugs, and on the relationship of intensity and duration of treatment to carcinogenicity has been limited, however, and has been almost entirely in relation to leukaemia, not solid cancer risk. The Royal Marsden Hospital (RMH) has been involved in intensive treatment of Hodgkin's disease since the 1960s, when this therapy was first used, and its database of patients treated, makes available more than 25 years of follow-up. A previous publication described second malignancies up to the end of 1983 in patients first treated before 1979 (Colman et al, 1988). The present analyses extend the numbers and length of follow-up for Hodgkin's disease patients at the RMH, and also extend the range of analyses of risks in these patients, especially with regard to solid cancer.

*Received 19 April 1996*
*Revised 20 June 1996*
*Accepted 23 July 1996*

*Correspondence to:* Professor A J Swerdlow

### MATERIALS AND METHODS

The RMH is one of the largest cancer treatment centres in the UK, and its computerized databases contain details of treatment and follow-up of Hodgkin's disease patients since 1963. For the present analyses we extracted data on all patients first treated from 1963 to March 1991 by one of the two treatment teams at the hospital, and from 1963 to February 1989 by the other team. We updated treatment and follow-up data on these patients to 30 June 1991. The treatment data included information on the cycles of treatment since diagnosis, including treatments before first attendance at the RMH. In some instances, the date of starting an individual cycle was known, and in others only the date of starting a course (of several cycles) of treatment; for the latter, we estimated the dates of individual cycles from information on the start date of the course, the number of cycles in the course and the mean duration per cycle. Regular follow-up of Hodgkin's disease patients at the RMH is conducted at least annually, unless the patient is transferred to another hospital, which then provided the available information. To check that follow-up for second cancers was complete, as well as examining case notes, we matched details of study subjects against cancer registration records for the South Thames Region, within which the RMH is situated, and extracted data on second cancers from this source. The regional cancer registry receives from the National Health Service Central Registers notification of second malignancies occurring anywhere in the country in patients resident in the registry region at the time of the initial Hodgkin's disease. Pathological review of second cancers was available where these had occurred while under follow-up at the RMH, but for second cancers in patients followed up elsewhere, the report from the current treatment centre was used. Patients resident outside the UK were excluded from the analyses because of incomplete follow up and the absence of appropriate data to calculate expected cancer incidence rate.

For each patient in the cohort, person–years at risk of second cancer by 5-year age group, sex and calendar year were calculated from date of first treatment to 30 June 1991, or to the date of death, second cancer incidence or loss to follow-up, if earlier. For analyses of time-dependent variables (e.g. interval since treatment and number of cycles of treatment), subjects were assigned at each stage of follow-up to the value of the variable applicable at that time. Observed numbers of cancers in the cohort were compared with expectations calculated by multiplying the person–years at risk in the cohort in each age, sex and calendar year category by the corresponding cancer registration rates in the general population of the South Thames region. As registration statistics were not available in computer-readable form before 1971 or after 1989, we applied 1971 data to give expecteds for 1963–71 and 1989 data to give expecteds for 1989–91. Site of second cancers was coded to the International Classification of Diseases (ICD) (WHO, 1978) revisions in force in England and Wales at the time of occurrence: ICD7 for 1963–67, ICD8 for 1968–78, and ICD9 for 1979 onwards. We reallocated the data coded to the earlier revisions ('bridge-coded' the data) to the ICD9 categories shown in Table 1. Standardized incidence ratios (SIRs) were then calculated as the ratio of observed to expected numbers of cancers. Ninety-five per cent confidence interval estimates for the SIRs were calculated using a likelihood-based method (Clayton and Hills, 1993). Site-specific absolute excess risks of second cancer were calculated by subtracting expected from observed numbers and dividing by person–years at risk. Cumulative (actuarial) probabilities of second cancer were calculated by the method of Kaplan and Meier (1958).

To compare risks in different treatment groups, with adjustment for possible confounding variables such as age at first treatment or number of cycles of treatment, relative risks were calculated by Cox regression (Cox, 1972). All significance levels are two-sided.

### RESULTS

A total of 1039 patients (649 men, 390 women) met the study criteria and were included in the cohort. Most (79%) were aged under 45 years at first treatment, ranging from 3 to 84 years. During follow-up 77 patients developed a second malignancy and, of the remainder, 332 died, 20 emigrated, four were lost to follow-up and 606 survived to the end of June 1991. The follow-up was for 9516 person–years in total, an average of 9.1 years per cohort member.

As well as 77 second malignancies during follow-up, there were two *in situ* tumours of the cervix and two of the skin, one benign parotid tumour and one brain tumour of unspecified nature, which

**Table 1** Relative risks of second primary cancer of selected sites

| | ICD9 Site | No. | SIR (95% CI) | Absolute excess risk per 10 000 person–years |
|---|---|---|---|---|
| 141–9 | Tongue, mouth and pharynx | 2 | 5.7 (0.9–17.5) | 1.7 |
| 151 | Stomach | 4 | 4.0 (1.2–9.3)* | 3.2 |
| 154 | Colon | 2 | 1.4 (0.2–4.4) | 0.6 |
| 162 | Lung | 15 | 3.8 (2.2–6.0)*** | 11.6 |
| 170 | Bone | 2 | 26.5 (4.4–81.8)** | 2.0 |
| 171 | Soft tissue | 2 | 16.9 (2.8–52.3)** | 2.0 |
| 172 | Malignant melanoma | 2 | 4.0 (0.7–12.2) | 1.6 |
| 173 | Non-melanoma skin cancer | 10 | 3.9 (1.9–6.8)*** | 7.8 |
| 175 | Breast (female) | 5 | 1.8 (0.7–3.9) | 2.4 |
| 184 | Prostate | 2 | 2.1 (0.4–6.5) | 1.1 |
| 193 | Thyroid | 1 | 8.2 (0.5–36.0) | 0.9 |
| 195–9 | Unknown primary | 3 | 3.5 (0.9–9.1) | 2.3 |
| 200, 202 | Non-Hodgkin's lymphoma | 3 | 4.6 (1.2–12.0)* | 2.5 |
| 204–8 | All leukaemia | 13 | 23.5 (12.9–38.6)*** | 13.1 |
| 204.0, 204.2–208.9 | Leukaemia other than chronic lymphoid leukaemia (CLL) | 12 | 31.3 (16.7–52.4)*** | 12.2 |
| 204.1 | CLL | 1 | 5.9 (0.3–25.8) | 0.9 |
| 140–172, 174–200, 202–208 | All malignancies except non-melanoma skin cancer and Hodgkin's disease[a] | 67 | 3.3 (2.6–4.2)*** | 49.2 |

**P*<0.05; ***P*<0.01; ****P*<0.001. [a]Includes, apart from the tumours above, one cancer of the lip, one of the oesophagus, one rectum, one pancreas, one unspecified digestive, one cervix, one corpus uteri, one testis, two bladder, one brain.

*© Cancer Research Campaign 1997*

**Table 2** Cumulative (actuarial) risks of second primary cancer

| Years since first treatment | Lung cancer % probability (95% CI) | All other solid tumours excluding non-melanoma skin cancer % probability (95% CI) | Leukaemia % probability (95% CI) | All malignancies except Hodgkin's disease and non-melanoma skin cancer % probability (95% CI) |
|---|---|---|---|---|
| 5 | 0.2 (0.1–0.9) | 1.9 (1.1–3.2) | 0.6 (0.3–1.5) | 2.7 (1.8–4.1) |
| 10 | 1.3 (0.6–2.6) | 3.6 (2.4–5.3) | 1.9 (1.1–3.4) | 6.9 (5.2–9.2) |
| 15 | 3.0 (1.7–5.1) | 5.2 (3.5–7.6) | 1.9 (1.1–3.4) | 10.3 (7.9–13.3) |
| 20 | 3.4 (2.0–5.8) | 8.3 (5.5–12.5) | 2.3 (1.3–4.2) | 14.0 (10.6–18.4) |

**Table 3** Relative risks of second primary cancer by treatment group

| Treatment group | Lung cancer | | All other solid cancers, excluding non-melanoma skin cancer | | Leukaemia | | All malignancies except Hodgkin's disease and non-melanoma skin cancer | |
|---|---|---|---|---|---|---|---|---|
| | No. | SIR (95% CI) | No. | SIR (95% CI) | No. | SIR (95% CI) | No. | SIR (95% CI) |
| Chemotherapy (person–years = 1531) | 3 | 3.9 (1.0–10.0) | 4 | 1.6 (0.5–3.6) | 5 | 50.4 (18.1–108.3)*** | 12 | 3.3 (1.8–5.6)*** |
| Radiotherapy (person–years = 3660) | 7 | 4.2 (1.8–8.1)** | 14 | 2.2 (1.2–3.6)** | 0 | – | 22 | 2.6 (1.6–3.8)*** |
| Combined (person–years = 4325) | 5 | 3.2 (1.2–6.9)* | 18 | 3.0 (1.8–4.7)*** | 8 | 34.9 (16.0–65.0)*** | 33 | 4.1 (2.8–5.6)*** |
| All treatments (person–years = 9516) | 15 | 3.8 (2.2–6.0)*** | 36 | 2.4 (1.7–3.3)*** | 13 | 23.4 (12.9–38.6)*** | 67 | 3.3 (2.6–4.2)*** |

*$P$<0.05; **$P$<0.01; ***$P$<0.001.

were not included in the analyses. One patient had a third cancer – pancreatic cancer after bladder cancer – and this too was not included in the analyses. No second cancers occurred in the first 6 months after treatment, suggesting that none had been incident but undetected before Hodgkin's disease treatment started, and were detected as a consequence of it. Table 1 shows site-specific relative risks of second cancer in the cohort. There was a more than threefold raised risk of cancer overall, with significantly raised risks of lung, bone, soft tissue and non-melanoma skin cancers, non-Hodgkin's lymphoma and acute and non-lymphoid leukaemia (ANLL). Two-thirds of the absolute excess risk of cancer in the cohort was due to solid tumours (Table 1), and one-third to lymphohaematopoietic malignancies. In the remainder of this paper, non-lung solid cancers are generally grouped because of small numbers at each site; non-melanoma skin cancers are omitted from the risk calculations because the data on expected rates are less satisfactory than for other sites (so that the all malignancy analyses are based on the remaining 67 cases); and all leukaemias, rather than ANLL separately, are analysed because reference rates are incomplete for ANLL, as a proportion of leukaemias recorded in the cancer registration data are of unknown subtype.

Table 2 shows cumulative risks of second cancer in the cohort. For all cancers except non-melanoma skin cancer, there was a cumulative risk of 14% at 20 years, mainly reflecting the risk of solid tumours (11%).

There were significantly raised risks of cancer overall for patients treated with each modality (Table 3), but risk was greatest for those given combined treatment (i.e. chemotherapy plus radiotherapy). Lung cancer risks were three- to fourfold raised in each treatment group, significantly after radiotherapy and combined modalities, and borderline significantly ($P$=0.05) after chemotherapy, based on a smaller number of person–years. Non-lung solid malignancy risks were also raised in each group, although significant only in the two larger groups – radiotherapy and combined-modality treatment. The relative risk for all solid cancers after radiotherapy alone was 2.6 (1.7–3.9; $P$<0.001), after chemotherapy alone 2.1 (0.9–4.1; $P$=0.08) and after combined modality treatment 3.1 (2.0–4.5; $P$<0.001). Leukaemia risks were greatly raised in chemotherapy and combined modality-treated patients, but no cases occurred in patients treated solely with radiotherapy. Among non-lung solid tumours, there were significantly raised risks of lip cancer (SIR=82.9) in radiotherapy patients, stomach (SIR=7.7), soft tissue (SIR=38.6) and non-melanoma skin (SIR=5.8) cancers in mixed-modality patients, and tongue cancer (SIR=78.3) in chemotherapy patients.

Adjusting the treatment group comparisons in Table 3 for sex, age at first treatment, year of first treatment and duration since first treatment by Cox regression (not shown in table), increased the risks of solid cancers and of all cancers after chemotherapy alone compared with after combined modalities or radiotherapy alone: for all solid cancers, the adjusted relative risks for these three treatment groups were 1.0, 1.0 (0.4–2.4) and 1.0 (0.4–2.4) respectively, and for all cancers they were 1.0, 0.8 (0.4–1.6) and 0.6 (0.3–1.2). For leukaemia, the risk for combined-modality treatment compared with chemotherapy alone was 0.4 (0.1–1.3), and no cases occurred in patients treated solely with radiotherapy.

The relative risk of second malignancy overall was non-significantly greater in men than women ($P$=0.6) (Table 4). There was a non-significantly greater risk of non-lung solid cancers in men than women, and of lung cancer and leukaemia in women than

*© Cancer Research Campaign 1997*

**Table 4** Relative and absolute excess risks of second cancer by sex, and age at first treatment

| | Lung cancer | | | All other solid cancers, excluding non-melanoma skin cancer | | | Leukaemia | | | All malignancies except Hodgkin's disease and non-melanoma skin cancer | | |
|---|---|---|---|---|---|---|---|---|---|---|---|---|
| | No. | SIR (95% CI) | AR[a] | No. | SIR (95% CI) | AR[a] | No. | SIR (95% CI) | AR[a] | No. | SIR (95% CI) | AR[a] |
| Sex | | | | | | | | | | | | |
| Male ($n$=649) | 10 | 3.1 (1.6–5.5)** | 12.0 | 21 | 2.8 (1.8–4.2)*** | 23.7 | 8 | 21.7 (9.9–40.5)*** | 13.4 | 41 | 3.5 (2.5–4.7)*** | 51.5 |
| Female ($n$=390) | 5 | 6.3 (2.3–13.6)** | 11.0 | 15 | 2.1 (1.2–3.3)* | 20.2 | 5 | 26.8 (9.6–57.6)*** | 12.6 | 26 | 3.0 (2.0–4.4)*** | 45.6 |
| Age (years) | | | | | | | | | | | | |
| <25 ($n$=377) | 2 | 41.4 (6.9–127.8)*** | 5.0 | 9 | 7.4 (3.5–13.3)*** | 19.7 | 2 | 24.0 (4.0–74.2)** | 4.9 | 13 | 8.9 (4.9–14.7)*** | 29.3 |
| 25–44 ($n$=447) | 4 | 6.2 (1.9–14.4)** | 8.1 | 13 | 2.8 (1.5–4.6)** | 20.2 | 7 | 43.6 (18.8–84.4)*** | 16.5 | 24 | 4.2 (2.7–6.1)*** | 44.1 |
| 45–54 ($n$=86) | 5 | 5.3 (1.9–11.4)** | 57.1 | 5 | 1.8 (0.6–3.8) | 31.0 | 2 | 22.5 (3.7–69.4)** | 26.9 | 13 | 3.3 (1.8–5.4)*** | 126.6 |
| ≥55 ($n$=129) | 4 | 1.7 (0.5–3.9) | 22.6 | 9 | 1.5 (0.7–2.6) | 39.6 | 2 | 9.0 (1.5–27.8)* | 24.6 | 17 | 1.9 (1.1–2.9)* | 110.2 |
| $X^2_1$trend | | 8.96** | | | 9.95** | | | 2.58 | | | 16.36*** | |

[a]Absolute excess risk per 10 000 person–years. *$P$<0.05; **$P$<0.01; ***$P$<0.01.

**Table 5** Relative and absolute excess risks of second primary cancer by attained age

| Age (years) | Lung cancer | | | All other solid cancers except non-melanoma skin cancer | | | Leukaemia | | | All malignancies except non-melanoma skin cancer and Hodgkin's disease | | |
|---|---|---|---|---|---|---|---|---|---|---|---|---|
| | No. | SIR (95% CI) | AR[a] | No. | SIR (95% CI) | AR[a] | No. | SIR (95% CI) | AR[a] | No. | SIR (95% CI) | AR[a] |
| <25 (person–years = 1772) | 1 | 354 (20.2–1559)** | 5.6 | 5 | 25.5 (9.1–54.7)*** | 27.1 | 1 | 30.9 (1.8–136)* | 5.5 | 7 | 27.5 (11.8–53.2)*** | 38.0 |
| 25–44 (person–years = 5264) | 2 | 10.0 (1.7–30.8)* | 3.4 | 9 | 3.0 (1.4–5.3)** | 11.1 | 5 | 36.0 (12.9–77.5)*** | 9.2 | 16 | 4.4 (2.6–7.0)*** | 23.5 |
| 45–54 (person–years = 1205) | 5 | 10.1 (3.6–21.7)*** | 37.4 | 7 | 2.7 (1.2–5.3)* | 36.9 | 5 | 64.1 (23.0–138)*** | 40.8 | 18 | 5.5 (3.3–8.4)*** | 122.2 |
| ≥55 (person–years = 1275) | 7 | 2.1 (0.9–4.1) | 29.0 | 15 | 1.7(1.0–2.7) | 47.1 | 2 | 6.5 (1.1–20.2)* | 13.3 | 26 | 2.0 (1.3–2.8)** | 101.3 |
| $X^2_1$trend | | 11.0*** | | | 10.4** | | | 3.8 | | | 21.3*** | |

[a]Absolute excess risk per 10 000. *$P$<0.05; **$P$<0.01; ***$P$<0.001.

**Table 6** Relative risks of second cancer by duration since first treatment

| Duration (years) | Lung cancer | | All other solid cancers, excluding non-melanoma skin cancer | | Leukaemia | | All malignancies except Hodgkin's disease and non-melanoma skin cancer | |
|---|---|---|---|---|---|---|---|---|
| | No. | SIR (95% CI) | No. | SIR (95% CI) | No. | SIR (95% CI) | No. | SIR (95% CI) |
| 0–4 (person–years = 4078) | 2 | 1.3 (0.2–4.0) | 14 | 2.8 (1.6–4.5)** | 5 | 23.6 (8.5–50.8)*** | 21 | 2.9 (1.9–4.4)*** |
| 5–9 (person–years = 2684) | 6 | 5.7 (2.3–11.6)*** | 9 | 2.3 (1.1–4.2)* | 7 | 47.0 (20.2–90.9)*** | 24 | 4.6 (3.0–6.6)*** |
| 10–14 (person–years = 1656) | 6 | 7.8 (3.1–15.8)*** | 5 | 1.7 (0.6–3.7) | 0 | – | 12 | 3.0 (1.6–5.1)** |
| ≥15 (person–years = 1098) | 1 | 1.6 (0.1–7.0) | 8 | 2.7 (1.2–5.1)* | 1 | 11.2 (0.6–49.4) | 10 | 2.6 (1.3–4.6)** |

*$P$<0.05; **$P$<0.01; ***$P$<0.001.
For each cancer site catergory shown, $X^2_1$ trend not significant.

men. Relative risks of second cancer were greatly dependent on age at first treatment (Table 4). The relative risk for cancer overall was 8.9 in patients treated before age 25, compared with less than 2 in those treated at ages 55 and above. The age gradient was marked and highly significant for solid cancers ($P$<0.001), and separately for lung cancer and other solid cancers, but for leukaemia the greatest relative risk was for treatment at ages 25–44, with a diminishing relative risk for ages thereafter. For breast cancer (not shown in table), the relative risks were 7.8 (1.3–24.2) for first treatment before age 25 years, [32.8 (5.5–101.3) for first treatment before age 20] and 1.2 (0.3–3.1) for first treatment at ages 25 and above. The 20-year cumulative risk of breast cancer for women

**Table 7** Relative risks of second primary cancer in relation to specific chemotherapy treatments

| Treatment | Solid cancers | | Leukaemia | | All malignancies except non-melanoma skin cancer and Hodgkin's disease | |
|---|---|---|---|---|---|---|
| | No. | RR[a] (95% CI) | No. | RR[a](95% CI) | No. | RR[a] (95% CI) |
| MOPP[b] (person–years = 1406) | 9 | 1.0 | 2 | 1.0 | 11 | 1.0 |
| MOPP + other[c] (person–years = 399) | 0 | – | 5 | 8.0 (1.2–53.6)* | 5 | 1.8 (0.6–5.5) |
| ChlVPP (person–years = 2751) | 13 | 1.1 (0.3–4.2) | 3 | 2.3 (0.2–22.7) | 18 | 1.6 (0.5–4.6) |
| ChlVPP + other[d] (person–years = 471) | 2 | 0.5 (0.05–4.5) | 1 | 1.4 (0.05–39.6) | 3 | 0.7 (0.1–4.3) |

*$P<0.05$ [a]Adjusted for sex, age at first treatment, year of first treatment, duration since first treatment, number of cycles of chemotherapy and radiotherapy. [b]Baseline group for calculation of relative risks. [c]Including MOPP + ChlVPP. [d]Excluding ChlPP + MOPP. One second malignancy occurred in patients treated solely with chemotherapy regimens other than those in the table (person–years = 469).

**Table 8** Relative risks of second primary cancer in relation to number of cycles of chemotherapy

| No. of cycles | Solid cancers | | Leukaemia | | All malignancies except non-melanoma skin cancer and Hodkin's disease | |
|---|---|---|---|---|---|---|
| | No. | RR[a] (95% CI) | No. | RR[a] (95% CI) | No. | RR[a] (95% CI) |
| 1–6 (person–years = 3028) | 9 | 0.4 (0.1–1.3) | 0 | – | 11 | 0.2 (0.1–0.6)** |
| 7–12 (person–years = 1646) | 11 | 1.1 (0.4–3.4) | 5 | 0.4 (0.1–1.3) | 16 | 0.7 (0.3–1.6) |
| >12[b] (person–years = 822) | 5 | 1.0 | 6 | 1.0 | 11 | 1.0 |
| $\chi^2_1$ trend | | 3.4 | | 12.9*** | | 10.11** |

**$P<0.01$ ***$P<0.001$ [a]Adjusted for sex, age at first treatment, year of first treatment, duration since first treatment, and radiotherapy. [b]Baseline group for calculation of relative risks.

first treated before age 20 years was 4.1% (0.9–16.9), for those first treated before age 25 years was 2.1% (0.5–9.0) and for those first treated at age 25 years or more was 2.1% (0.7–6.5). All of the breast cancers, except one in a woman first treated at age 61 with chemotherapy plus radiotherapy to a tonsil, were after mantle radiotherapy alone or, in one instance, with chlorambucil, vinblastine, procarbazine and prednisone (ChlVPP).

When relative risks were considered in relation to attained age (Table 5), there were large, highly significant trends of greater risk at younger ages for all cancers and solid cancers, and a borderline significant trend ($P$=0.05) for leukaemia. Absolute excess risks were greater at ages 45 and above than at ages younger than this but, otherwise, did not show a consistent gradient with age. In treatment-specific analyses (not shown in table), aggregating the categories used in Table 5 when necessary because of small numbers, there were significant trends of increasing relative risk with younger age for all cancers and for solid cancers, but not for leukaemia, after radiotherapy and after combined-modality therapy. For patients treated solely with chemotherapy, there was a significant trend of greater risk with younger age ($P$=0.002) for all cancers, relative risks for leukaemia of 79.6 (13.2–245.9; $P<0.001$) for ages under 45 and 40.5 (10.1–105.0; $P<0.001$) for ages 45 and above, and for solid cancers there were relative risks of 4.3 (0.7–13.4) and 1.7 (0.6–3.8) for these age groups respectively. The relative risk of breast cancer for women aged under 30 years was 43.0 (7.1–132.7; $P<0.001$), compared with 1.1 (0.3–2.9) for women aged 30 years or more.

Relative risks of solid cancers remained increased 15 and more years after first treatment (Table 6), and there was no significant trend in risk with duration since first treatment. The leukaemia relative risk reached a peak at 5–9 years after first treatment, and then diminished greatly.

To explore further the relation of chemotherapy to risk of second cancer we divided patients who had received any chemotherapy into five groups according to the chemotherapeutic agents received (Table 7) and into three groups by the number of cycles of treatment (Table 8). We included patients in these analyses irrespective of whether they had received radiotherapy in addition to their chemotherapy, but we adjusted for whether or not radiotherapy had been given. The analyses were based on 745 patients treated with chemotherapy; 58 patients were excluded for whom there was insufficient information on dates of cycles of chemotherapy. In categorizing the different treatment regimens, we included with MOPP (mustine, vincristine, procarbazine and prednisone), treatments in which vinblastine had been substituted for vincristine, and with ChlVPP, treatments with vincristine substituted for vinblastine. The most common regimens included under 'other' chemotherapy were VEEP (vincristine, etoposide, epirubicin, prednisolone), which had been received by 114 patients, melphalan plus BCNU plus etoposide (55 patients), and ABVD (doxorubicin, bleomycin, vinblastine, dacarbazine) (47 patients). Leukaemia and solid cancer risks were not significantly greater for patients treated with ChlVPP alone than for those treated with MOPP alone (Table 7). Only one second cancer

 © Cancer Research Campaign 1997

occurred in patients treated with chemotherapy that included neither MOPP nor ChlVPP; 0.84 were expected in these patients on the basis of South Thames general population cancer rates.

Most patients treated with nitrosoureas had also been treated with MOPP or ChlVPP, but analyses of risk in relation to whether nitrosoureas had ever been administered showed a large raised risk of leukaemia: the relative risk of leukaemia for patients ever-receiving nitrosoureas compared with those who received MOPP alone was 38.5 (3.9–384.3), adjusted as in Table 7. There was no raised risk of solid cancers after nitrosoureas compared with MOPP alone, but only two solid cancers occurred after nitrosourea treatment.

In the analyses of risk in relation to number of cycles of treatment (Table 8), there was a significant ($P$=0.002) trend of greater all-cancer risk with more cycles of treatment, which arose from a significant ($P$=<0.001) trend for leukaemia (which occurred only in patients receiving more than 6 cycles) and a non-significant ($P$=0.07) trend in the same direction for solid malignancy.

### DISCUSSION

The range of sites for which second cancer risk was raised in the present data accords with findings from other cohorts of patients treated for Hodgkin's disease (Kaldor et al, 1987; Tucker et al, 1988; Henri-Amar, 1992; Swerdlow et al, 1992; van Leeuwen et al, 1994*a*). As in several recent studies where long-term follow-up is available (Tucker et al, 1988; Henri-Amar, 1992; Swerdlow et al, 1992; van Leeuwen et al, 1994*a*; Boivin et al, 1995) it is clear in the present data that leukaemia risk is an early consequence of chemotherapy, but one that diminishes beyond 10 years from initial treatment. Solid cancer risks, however, continued to be significantly raised even 15 years after treatment. In the long-term, the solid cancer risk is the major second malignancy threat to Hodgkin's disease patients. The relative risk of non-Hodgkin's lymphoma in our data was similar to that in a large US study (Boivin et al, 1995), although lower than in several other recent studies (Henri-Amar, 1992; Swerdlow et al, 1992; van Leeuwen et al, 1994*a*). We have checked that cases of non-Hodgkin's lymphoma were not omitted from the analysis, and in the process found five more cases currently excluded because they occurred in overseas residents or occurred after the end of the analysed follow-up. The cumulative risks of second malignancy need to be interpreted cautiously; because they take no account of the particular background population risks of malignancy 'expected' in the study area catchment population at the time of the follow-up, they cannot directly be generalized to, or compared with, such risks in other series of patients with Hodgkin's disease. Similarly, comparisons with other series need to take account of the age and sex distributions of the different patient groups, which are powerful influences on cumulative risk. It should also be noted that the cumulative risks are *conditional* on remaining cancer free and alive up to that point; they are not the percentage chance of developing the second cancer for a patient at the start of treatment. As they involve competing risks, the cumulative risks for particular cancers cannot be summed to give the cumulative risk for these cancers in total (Clayton and Hills, 1993).

It is known from several studies (Boivin and O'Brien, 1988; Tucker et al, 1988; Swerdlow et al, 1992; van Leeuwen et al, 1994), and unsurprising in the light of the findings in other radiation-exposed cohorts such as atomic bomb survivors (National Research Council, 1990), that solid cancer risks can be increased by radiotherapy. Relative risks in patients treated with combined modalities are also raised, and appear to be fairly similar to those for radiotherapy alone. The risks after chemotherapy alone are much less clear, however. There have been relatively small numbers of person–years of long-term follow-up after chemotherapy without radiotherapy in most cohorts yet published, and until recently the power of such cohorts has been low and increased risk of solid cancers has not been seen (Boivin and O'Brien, 1988; Young et al, 1990). Four recent studies have shown increased solid cancer risk after chemotherapy (Kaldor et al, 1992; Swerdlow et al, 1992; Biti et al, 1994; Boivin et al, 1995) while others did not (Abrahamsen et al, 1993; van Leeuwen et al, 1994*b*). The reason for the difference in findings is not obvious. The current results add to evidence for an effect of chemotherapy on solid malignancy risks. The raised risk in our data related entirely to MOPP and ChlVPP treatments, but we had far too few person–years of follow-up after any other specific treatment to determine risks for these other regimens. Risk after ChlVPP was similar (non-significantly greater) to that after MOPP in the present study. In Boivin's (1995) study, too, based on larger numbers, risks after mustine and chlorambucil were similar. In British National Lymphoma Investigation (BNLI) data (Swerdlow et al, 1992), the solid cancer relative risk was similar for mustine-containing and chlorambucil-containing combinations, after adjustment for confounders. An effect of chemotherapy on solid cancer risk is likely to be, at least to some extent, site specific. We had too few second cancers to separate individual sites other than lung, but for this site the borderline significant raised risk in our data in relation to chemotherapy accords with significant raised risks of this site in two previous studies (Kaldor et al, 1992; Swerdlow et al, 1992), although not in others (van Leeuwen et al, 1994*b*; Boivin et al, 1995).

Information on solid cancer risk in relation to dosage would help to clarify the possible aetiological role of chemotherapy for solid malignancies, but there have been few data published on this. In Kaldor's (1992) study, which was solely of lung cancer after Hodgkin's disease, risk was not associated with the number of cycles of MOPP. In BNLI data (Swerdlow et al, 1992) the number of courses of treatment showed no relation to solid malignancy risk, but the great majority of the exposure was in the one-course category. In the present data there was a trend in the direction of greater risk with more cycles, although not quite significant ($P$=0.07).

As in several previous analyses (Pedersen-Bjegaard et al, 1987; van der Velden et al, 1988; Henri-Amar et al, 1989; Kaldor et al, 1990; van Leeuwen et al, 1994*b*), risk of leukaemia increased dramatically with dose or number of cycles of treatment and, indeed, there were no leukaemias in the present study in patients treated with only six cycles (one standard course) or less. As the high risk of leukaemia after chemotherapy became clear, various alternatives to the most used original combination, MOPP, have been introduced, which might lead to a lower risk of leukaemia, but studies have often had too few patients with any particular other treatment to divide their analyses further than MOPP and 'other' chemotherapy. In the largest published analysis (Boivin et al, 1995), leukaemia risk after chlorambucil was over twice that after mustine, which is similar to the present results. Kaldor et al (1990) found a lower risk after chlorambucil–procarbazine combinations, but a greater risk after chlorambucil without procarbazine, than after mustine–procarbazine combinations, although based on few cases for the first two categories. Glicksman et al (1982) found a large raised risk of leukaemia, based on three cases, in patients receiving chlorambucil maintenance therapy but no

© Cancer Research Campaign 1997

mechlorethamine, and an SRR of 700 when mechlorethamine induction plus chlorambucil maintenance was employed. In BNLI data (Swerdlow et al, 1992), no leukaemias occurred after chlorambucil, but only 0.06 were expected. Chlorambucil is less acutely toxic and as effective as mustine in combination chemotherapy (Selby et al, 1990), but it would appear not to be a solution to the problem of carcinogenicity.

The decrease in relative risk of second cancers overall with increasing age at first treatment accords with several previous reports (Glicksman et al, 1982; Sont et al, 1992; Abrahamsen et al, 1993; Swerdlow et al, 1993). For leukaemia, previous studies (Glicksman et al, 1982; van der Velden et al, 1988; Abrahamsen et al, 1993; Swerdlow et al, 1993) agree with the present analyses that relative risks diminish beyond middle age, and this has been the case in analyses where ANLL or AML were analysed separately (Glicksman et al, 1982; van der Velden et al, 1988), but results have been variable with regard to the trend in risks at younger ages of first treatment.

Surprisingly, there are almost no previous data on relative risks of solid malignancies in relation to age at first treatment except for breast cancer. For this tumour, previous studies concur that there is a large relative risk in follow-up beyond 15 years for women treated at young ages with chest radiotherapy (Hancock et al, 1993; van Leeuwen et al, 1994*a*; Boivin et al, 1995), but have differed on whether (Hancock et al, 1993) or not (van Leeuwen et al, 1994*a*; Boivin et al, 1995) there is a raised risk before 15 years of follow-up; in our data all of the breast cancers occurred before 15 years from first treatment. Careful clinical surveillance to detect breast cancers early is needed in women treated young with mantle radiotherapy. The only previous data on relative risks by age at first treatment for solid cancers more generally appear to be those from the BNLI (Swerdlow et al, 1993), which showed a significant trend of increasing relative risk with decreasing age (although less steep than in the present study) for non-lung solid cancers, and a non-significant trend in the same direction (except that no cases occurred under age 25) for lung cancer. The sole previous analyses by attained age appear to be those on breast cancer risk by Hancock et al (1993). These showed, as in the present data, a large relative risk for women treated under age 30 years. Our data set was not large enough to distinguish the separate contributions of age at first treatment and attained age to solid cancer risk, but the distinction is important to make. A relation of relative risk to young attained age would imply a decreasing relative risk of second malignancy for young patients as they grow older. On the other hand, an effect based on age at first treatment would imply, more worryingly, that relative risks will remain very high in these patients as they age. The results by duration since first treatment in our data and previously (Kaldor et al, 1987; Tucker et al, 1988; Henri-Amar, 1992; Swerdlow et al, 1992; van Leeuwen et al, 1994*a*) suggest that relative risks will not cease to be raised simply because of the passage of time, for at least 10–20 years after first treatment. If large relative risks remain as the patients grow older, they will translate into very large absolute risks as the background risks rise with age.

The risks of second malignancies are small compared with the benefits that intensive radiotherapy and chemotherapy have brought to the treatment of Hodgkin's disease but further monitoring and refinement of treatments are needed to reduce these adverse long-term side effects.

### ACKNOWLEDGEMENTS

We thank the Thames Cancer Registry for data on second cancers, Ms S Campbell, Ms J Nicholls and Mrs L Wickens for help in data extraction and Mrs E Middleton for secretarial help. The Epidemiological Monitoring Unit is funded by the Medical Research Council.

### REFERENCES

Abrahamsen JF, Andersen A, Hannisdal E, Nome O, Abrahamsen AF, Kvaløy S and Høst H (1993) Second malignancies after treatment of Hodgkin's disease: the influence of treatment, follow-up time, and age. *J Clin Oncol* **11:** 255–261

Biti G, Cellai E, Magrini SM, Papi MG, Ponticelli P and Boddi V (1994) Second solid tumors and leukemia after treatment for Hodgkin's disease: an analysis of 1121 patients from a single institution. *Int J Radiation Oncol Biol Phys* **29:** 25–31

Boivin J-F and O'Brien K (1988) Solid cancer risk after treatment of Hodgkin's disease. *Cancer* **61:** 2541–2546

Boivin J-F, Hutchison GB, Zauber AG, Bernstein L, Davis FG, Michel RP, Zanke B, Tan CTC, Fuller LM, Mauch P and Ultman JE (1995) Incidence of second cancers in patients treated for Hodgkin's disease. *J Natl Cancer Inst* **87:** 732–741

Clayton D and Hills M (1993) *Statistical Models in Epidemiology.* Oxford University Press: Oxford

Colman M, Easton DF, Horwich A and Peckham MJ (1988) Second malignancies and Hodgkin's disease – The Royal Marsden Hospital experience. *Radiother Oncol* **11:** 229–238

Cox DR (1972) Regression models and life-tables. *J R Stat Soc* (B) **34:** 187–202

Glicksman AS, Pajak TF, Gottlieb A, Nissen N, Stutzman L and Cooper MR (1982) Second malignant neoplasms in patients successfully treated for Hodgkin's disease: a Cancer and Leukemia Group B Study. *Cancer Treatment Rep* **66:** 1035–1044

Hancock SL, Tucker MA and Hoppe RT (1993) Breast cancer after treatment of Hodgkin's disease. *J Natl Cancer Inst* **85:** 25–31

Henri-Amar M on behalf of the International Database on Hodgkin's Disease (IDHD) (1992) Second cancer after the treatment for Hodgkin's disease: a report from the International Database on Hodgkin's Disease. *Ann Oncol* **3** (Suppl. 4): S117–S128

Henri-Amar M, Pellae-Cosset B, Bayle-Weisgerber C, Hayat M, Cosset JM, Carde P and Tubiana M (1989) Risk of secondary acute leukemia and preleukemia after Hodgkin's disease: the Institut Gustave-Roussy experience. *Recent Results Cancer Res* **117:** 270–283

Kaldor JM, Day NE, Band P, Choi NW, Clarke EA, Coleman MP, Hakama M, Koch M, Langmark F, Neal FE, Pettersson F, Pompe-Kirn V, Prior P and Storm H (1987) Second malignancies following testicular cancer, ovarian cancer and Hodgkin's disease: an international collaborative study among cancer registries. *Int J Cancer* **39:** 571–585

Kaldor JM, Day NE, Clarke EA, Van Leeuwen FE, Henry-Amar M, Fiorentino MV, Bell J, Pedersen D, Band P, Assouline D, Koch M, Choi W, Prior P, Blair V, Langmark F, Kirn VP, Neal F, Peters D, Pfeiffer R, Karjalainen S, Cuzick J, Sutcliffe SB, Somers R, Pellae-Cosset B, Pappagallo GL, Fraser P, Storm H and Storall M (1990) Leukemia following Hodgkin's disease. *N Engl J Med* **322:** 7–13

Kaldor JM, Day NE, Bell J, Clarke EA, Langmark F, Karjalainen S, Band P, Pedersen D, Choi W, Blair V, Henri-Amar M, Prior P, Assouline D, Pompe-Kirn V, Cartwright RA, Koch M, Arslan A, Fraser P, Sutcliffe SB, Host H, Hakama M and Storall M, (1992) Lung cancer following Hodgkin's disease: a case-control study. *Int J Cancer* **52:** 677–681

Kaplan EL and Meier P (1958) Non-parametric estimation from incomplete observations. *J Am Stat Assoc* **53:** 457–481

National Research Council (1990) *Health Effects of Exposure to Low Levels of Ionizing Radiation.* BEIR V. National Academy Press: Washington DC

Pedersen-Bjegaard J, Specht L, Larsen SO, Ersbøll J, Struck J, Hansen MM, Hansen HH, Nissen NI (1987) Risk of therapy-related leukaemia and preleukaemia after Hodgkin's disease. Relation to age, cumulative dose of alkylating agents, and time from chemotherapy. *Lancet* **2:** 83–88

Selby P, Patel P, Milan S, Meldrum M, Mansi J, Mbidde E, Brada M, Perren T, Forgeson G, Gore M, Smith I and McElwain T (1990) ChLVPP combination chemotherapy for Hodgkin's disease: long term results. *Br J Cancer* **62:** 279–285

© Cancer Research Campaign 1997

Sont JK, van Stiphout WAHJ, Noordijk EM, Molenaar J, Zwetsloot-Schonk JHM, Willemze R and Vandenbroucke JP (1992) Increased risk of second cancers in managing Hodgkin's disease: the 20-year Leiden experience. *Ann Hematol* **65:** 213–218

Swerdlow AJ, Douglas AJ, Vaughan Hudson G, Vaughan Hudson B, Bennett MH and MacLennan KA (1992) Risk of second primary cancers after Hodgkin's disease by type of treatment: analysis of 2846 patients in the British National Lymphoma Investigation. *Br Med J* **304:** 1137–1143

Swerdlow AJ, Douglas AJ, Vaughan Hudson G, Vaughan Hudson B and MacLennan KA (1993) Risk of second primary cancer after Hodgkin's disease in patients in the British National Lymphoma Investigation: relationships to host factors, histology and stage of Hodgkin's disease, and splenectomy. *Br J Cancer* **68:** 1006–1011

Tucker MA, Coleman CN, Cox RS, Varghese A and Rosenberg SA (1988) Risk of second cancers after treatment for Hodgkin's disease. *N Engl J Med* **318:** 76–81

Van der Velden JW, van Putten WLJ, Guinee VF, Pfeiffer R, van Leeuwen FE, van der Linden EAM, Vardomskaya I, Lane W, Durand M, Lagarde C, Hagemeister FB, Hagenbeek A and Eghbali H (1988) Subsequent development of acute non-lymphocytic leukemia in patients treated for Hodgkin's disease. *Int J Cancer* **42:** 252–255

Van Leeuwen FE, Klokman WJ, Hagenbeek A, Noyon R, van den Belt-Dusebout AW, van Kerkhoff EHM, van Heerde P and Somers R (1994*a*) Second cancer risk following Hodgkin's disease: a 20-year follow-up study. *J Clin Oncol* **12:** 312–325

Van Leeuwen FE, Chorus AMJ, van den Belt-Dusebout AW, Hagenbeek A, Noyon R, van Kerkhoff EHM, Pinedo HM and Somers R (1994b) Leukemia risk following Hodgkin's disease: relation to cumulative dose of alkylating agents, treatment with teniposide combinations, number of episodes of chemotherapy, and bone marrow damage. *J Clin Oncol* **12:** 1063–1073

World Health Organization (1978) *Manual of the International Statistical Classification of Diseases, Injuries, and Causes of Death.* WHO: Geneva

Young RC, Bookman MA, Longo DL (1990) Late complications of Hodgkin's disease management. *J Natl Cancer Inst Monogr* **10:** 55–60

© Cancer Research Campaign 1997